# Immunological control of skin development: from homeostasis to developmental pathologies

**DOI:** 10.3389/fimmu.2025.1685633

**Published:** 2025-11-25

**Authors:** Yutong Wei, Jing Gu, Zifan Zhao, Bin Gu

**Affiliations:** 1 State Key Laboratory of Oral and Maxillofacial Reconstruction and Regeneration, Key Laboratory of Oral Biomedicine Ministry of Education, Hubei Key Laboratory of Stomatology, School and Hospital of Stomatology, Wuhan University, Wuhan, China; 2 Department of Hemodialysis, The Affiliated Taian City Central Hospital of Qingdao University, Taian, China; 3 Department of Oral Implantology, School and Hospital of Stomatology, Wuhan University, Wuhan, China

**Keywords:** skin, immune, development, repair, interaction, pathology

## Abstract

The skin serves not only as a physical barrier but also as a dynamic immune organ, where immune cells orchestrate tissue morphogenesis, homeostasis, and repair. Recent advances have revealed that immune cells play pivotal roles during skin development, guiding processes such as vascular formation, epidermal stratification, and hair follicle morphogenesis. In this review, we integrate fundamental mechanistic studies based on mouse models with key clinical observations from human diseases to comprehensively elucidate the contributions of critical immune cell populations—including macrophages, Langerhans cells, dendritic cells, mast cells, and innate lymphoid cells—to normal skin development. We then explore how dysregulation of immune cell functions leads to aberrant skin morphogenesis, contributing to congenital disorders, autoimmune-mediated abnormalities, and fibrotic diseases. By integrating insights from developmental immunology and pathology, we highlight how deviations in immune regulation can disrupt skin architecture and function. Understanding these mechanisms provides a foundation for developing targeted strategies to modulate immune pathways for therapeutic skin regeneration. Future studies integrating spatial and single-cell technologies will further refine our knowledge of immune-tissue crosstalk in skin development and disease.

## 1 Introduction

The skin is the largest organ in mammals, functioning as the primary barrier between the body and its environment while contributing to homeostasis, sensory perception, and immune defense (1). Its formation is a highly coordinated developmental process, regulated with precise temporal and spatial control. This process requires intricate signaling and structural integration among diverse cell populations with distinct developmental origins and fates.

Historically, research on skin development has centered on epidermis-derived structural cells—such as keratinocytes and hair follicle stem cells—and mesoderm-derived dermal fibroblasts and the vascular network (2–5). In contrast, immune cells were traditionally regarded as postnatal responders, engaged primarily in pathogen defense and the maintenance of immune homeostasis.

Recent advances in developmental immunology have challenged this view. Innate immune cells (particularly macrophages) not only perform traditional immune functions but also regulate key aspects of human prenatal skin morphogenesis—including follicle formation, scar-free healing, and angiogenesis—through cross-cell interactions with epidermal cells, fibroblasts, and endothelial cells (6, 7). During embryogenesis, macrophages (8, 9), Langerhans cells (LCs) (10, 11), mast cells (MCs) (12, 13), and innate lymphoid cells (ILCs) (14, 15) colonize the skin and influence morphogenesis by secreting growth factors, modulating cell proliferation and apoptosis, and orchestrating extracellular matrix remodeling (**Table 1**).

**Table 1:** Key immune cells in normal skin development and homeostasis.

Immune cell	Key mediators/Cytokines	Target cells	Key function in development & homeostasis
Macrophages	IL-10, TGF-β, VEGF-A, PIGF	Keratinocytes; Fibroblasts; Endothelial Cells;	Debris clearance; Angiogenesis (vascular network formation); ECM remodeling; Regulating local inflammation;
Langerhans Cells (LCs)	TGF-β, Antigen (presentation)	T cells (in lymph node); Keratinocytes;	Maintenance of epidermal network; Antigen presentation; Establishment of immune tolerance;
Dermal Dendritic Cells (DCs)	Antigen (presentation)	T cells	Antigen presentation; Induction of immune tolerance (cDC2s); Cross-presentation (cDC1s);
Plasmacytoid DCs (pDCs)	Type I Interferons α(IFN-α)	T cells; NK cells	Accumulate post-birth to establish an “antiviral readiness state”; coordinate innate/adaptive immunity
Mast Cells (MCs)	Histamine; Tryptase; Chymase; TNF;	Dermal DCs; Blood Vessels; Nerves;	Regulate vascular permeability; Promote DC migration; Wound healing; Neuro-immune interaction.
Innate Lymphoid Cells (ILC1s)	IFN-γ; TNF-α	Macrophages; Keratinocytes;	barrier maturation; immune initiation;
Innate Lymphoid Cells (ILC3s)	IL-17A; IL-22; CCL3;	Keratinocytes; Macrophages; HFSCs;	Barrier integrity; Wound re-epithelialization; Hair follicle regeneration;

These immune activities are tightly regulated in both time and space. Perturbations—whether through aberrant activation or loss of function—can disrupt the balance of developmental signaling, leading to congenital skin malformations, barrier defects, or heightened susceptibility to inflammatory and fibrotic diseases (16, 17). Elucidating the physiological contributions of immune cells to skin development therefore offers critical insight into the pathogenesis of developmental immunodys regulation–associated skin disorders.

Beyond its relevance to disease, understanding immune–skin interactions in development have direct implications for regenerative medicine. Insights from developmental immunology may inform strategies for tissue engineering, wound healing, and therapeutic immune modulation to enhance regeneration. The emergence of high-resolution technologies—including single-cell transcriptomics, spatial transcriptomics, and 3D skin bioprinting—now enables detailed mapping of the cellular and molecular trajectories that underlie skin morphogenesis, providing unprecedented opportunities to decode immune–tissue crosstalk.

In this review, we synthesize current knowledge on the roles of immune cells in normal skin development and discuss how dysregulation of these processes drives pathology. We then highlight emerging therapeutic strategies that leverage developmental immunology to promote skin repair and regeneration.

## 2 Normal skin development

The skin is composed of three primary layers: the epidermis, dermis, and subcutaneous tissue. The epidermis, the outermost epithelial layer, is derived from the ectoderm and begins to differentiate early during embryogenesis, establishing its initial structural organization. It consists primarily of keratinocytes and intercellular lipids (18, 19). Based on differentiation status and functional properties, keratinocytes are organized into distinct strata, arranged from the inside outward as the stratum basale, stratum spinosum, stratum granulosum, stratum lucidum (present in thick skin), and stratum corneum.

In mammalian embryonic development, the epidermis initially exists as a single layer of cells, which then undergo proliferation, stratification, and keratinization to form a multilayered squamous epithelium with an effective barrier function (4, 20–22). Basal keratinocytes possess strong proliferative capacity, maintaining epidermal renewal and repair. As cells migrate outward, they progressively differentiate, ultimately forming the anucleate corneocytes of the stratum corneum, which together establish the skin barrier (23).

In addition to keratinocytes, the epidermis contains melanocytes, Merkel cells, and LCs (24, 25). These specialized cell types are essential for pigment production, mechanosensation, and immune surveillance. LCs, a unique subset of professional antigen-presenting cells (APCs), play a central role in maintaining local immune homeostasis. Their colonization and maintenance within the epidermis are regulated by multiple cytokines and chemokines (11, 26–28). Beyond LCs, murine epidermis also harbors two distinct T cell subsets: γδ T cells and CD8^+^ tissue-resident memory (TRM) cells. γδ T cells are a unique, innate-like T cell population that colonizes the murine epidermis in distinct waves during embryogenesis, establishing themselves as a permanent tissue-resident population before birth (29), whereas CD8^+^ TRM cells, composed of non-circulating memory T cells, typically appear following the resolution of cutaneous inflammation (30–32).

Beneath the epidermis lies the dermis, a mesoderm-derived connective tissue that provides structural thickness and mechanical strength to the skin. The dermis not only supplies mechanical and nutritional support to the epidermis, but also serves as the developmental niche for skin appendages—including hair follicles, sweat glands, sebaceous glands—as well as the cutaneous vasculature and neural network. Anatomically, it is divided into the papillary dermis and the reticular dermis. The papillary dermis, located directly beneath the epidermis, contains densely packed fibroblasts, capillaries, and immune cells, serving as a critical interface for epidermal–dermal signaling. The reticular dermis is rich in thick collagen bundles, larger blood vessels, major nerve trunks, and adipose tissue, conferring tensile strength and shock absorption (33).

Fibroblasts are the predominant stromal cells in the dermis. During early embryogenesis, they migrate into the cutaneous mesenchyme, producing and remodeling extracellular matrix (ECM) components—including collagen, elastin, and glycosaminoglycans—that are essential for maintaining skin architecture and elasticity. The dermis also houses diverse immune cell populations, including macrophages, MCs, dendritic cells (DCs), γδ T cells, and ILCs. These immune cells contribute to skin development by secreting chemokines (e.g., CCL2, CXCL12) and growth factors (e.g., VEGF, TGF-β), thereby regulating processes such as hair follicle morphogenesis, angiogenesis, and ECM remodeling (34–36). Notably, macrophages colonize the dermis during embryogenesis and are involved in debris clearance, fibroblast activation, and promotion of vascular development (37, 38). ILCs may facilitate coordinated epidermal–dermal development through the secretion of IL-13 and IL-5 (39, 40). Additionally, the maturation of dermal innervation is closely linked to the distribution of immune cells, suggesting a functional neuro–immune–skin axis during development (41, 42).

The subcutaneous tissue (hypodermis), located beneath the dermis, is a loose connective tissue layer that anchors the skin to underlying muscle. It is primarily composed of adipose tissue—clusters of adipocytes—along with blood vessels, lymphatic vessels, and a small number of fibroblasts and immune cells. In addition to its roles in energy storage, thermal insulation, and mechanical cushioning, the hypodermis contributes to skin homeostasis and immune regulation through the secretion of various cytokines (43).

**Figure 1:**
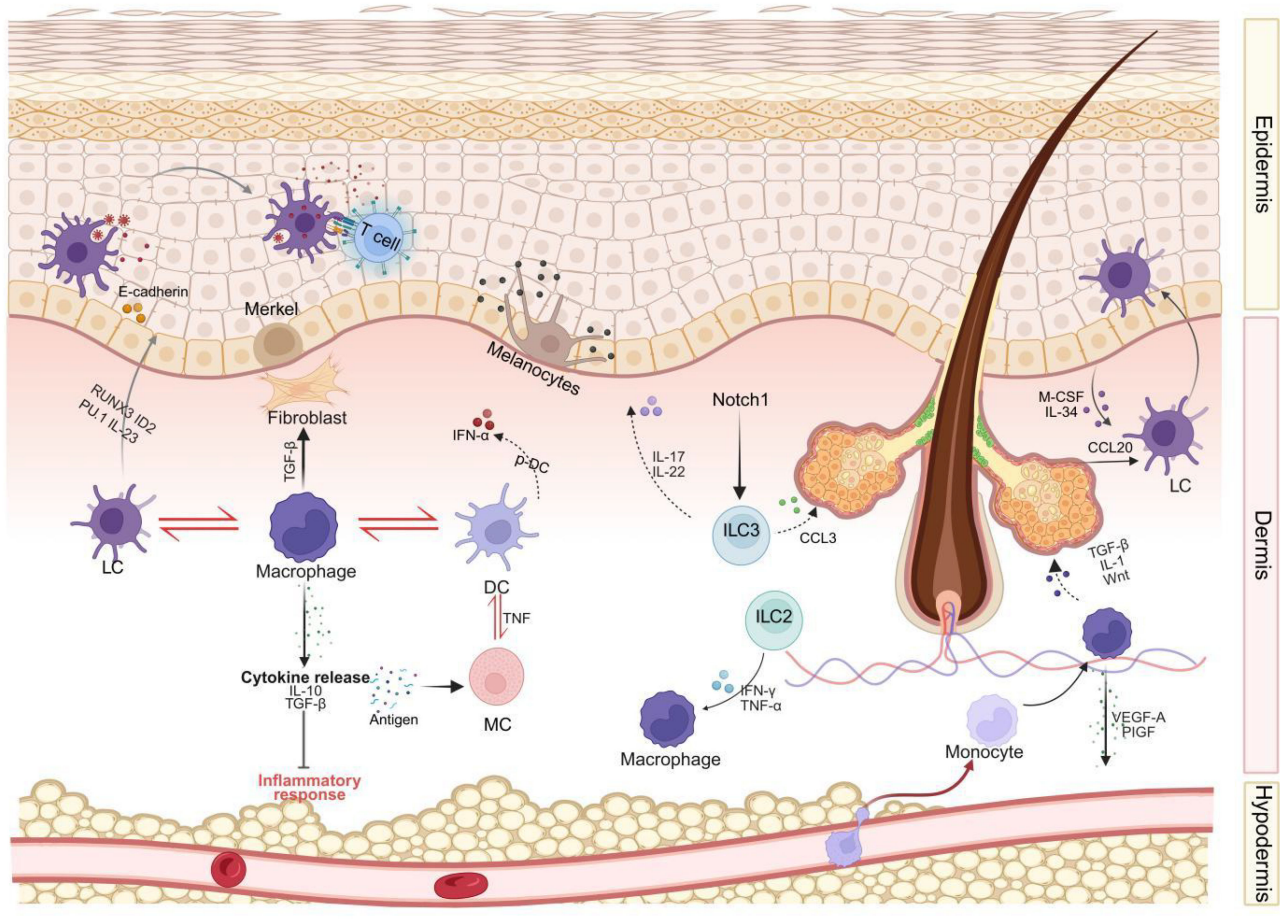
An integrated model of immune cell interactions during human skin development. This diagram integrates key signaling pathways, cell types, and interactions elucidated in human skin, encompassing different skin layers (epidermis, dermis, subcutaneous tissue). Langerhans cells (LCs) and T cells reside in the epidermal layer, interacting with keratinocytes, melanocytes, and Merkel cells. The dermis harbors a complex network comprising Langerhans cells (LCs), macrophages, dendritic cells (DCs), mast cells (MCs), innate lymphoid cells (ILCs), and fibroblasts. These cells interact both with each other (e.g., cross-talk between LCs-macrophages-DCs, DCs-MCs, macrophages-fibroblasts, and ILCs-keratinocytes) and with structural components such as hair follicles. Illustrated key signaling pathways include cytokine secretion (e.g., IL-10, TGF-β, VEGF released by macrophages; IL-17, IL-22, CCL3 released by ILC3) and chemokine secretion (e.g., CCL20 guiding Langerhans cells). Subcutaneous tissue and its vascular network support immune cell infiltration and are regulated by adipokines. LC, Langerhans cells; DC, dendritic cells; MC, mast cells; ILC, innate lymphoid cells; TNF-α, tumor necrosis factor-α; IFN-γ, interferon-γ; IL, interleukin; TNF, Tumor Necrosis Factor; TGF-β, transforming growth factor-β; VEGF, vascular endothelial growth factor; CCL, Chemokine (C-C motif) ligand; M-CSF, Macrophage colony-stimulating factor; PlGF, Placental Growth Factor; RUNX3, Runt-related transcription factor 3; ID2, Inhibitor of DNA binding 2.

During embryogenesis, subcutaneous adipose tissue arises from mesoderm-derived mesenchymal stem cells, which differentiate into adipocytes under defined signaling cues. These adipocytes progressively organize into lobules and expand rapidly during the perinatal and early postnatal periods. Adipose tissue secretes metabolic regulators such as leptin, adiponectin, and IL-6, influencing systemic metabolism, while also modulating the local immune microenvironment through immune mediators (44, 45). Immune cells—including macrophages, MCs, T cells, and ILCs—are integral to the hypodermis, where they participate not only in acute responses to external injury but also in maintaining local homeostasis, supporting hair follicle regeneration, and mediating chronic inflammation (**Figure 1**). The close association between subcutaneous adipose tissue and hair follicle growth, particularly during perinatal and pubertal stages, underscores the regulatory role of the hypodermis throughout skin development.

### 2.1 Macrophages

Macrophages are highly plastic cells of the hematopoietic system that are found in all tissues throughout the body. They are among the first immune cells to infiltrate the developing skin, originating from the yolk sac or liver early in embryogenesis. These cells migrate and settle within the skin, where they primarily function in phagocytosing apoptotic cells and tissue debris, thereby creating a clean microenvironment that supports the orderly construction of skin tissue. Not only do macrophages contribute to the initial stages of skin formation, but they also play a critical role throughout various phases of skin development.

During early skin development, macrophages regulate local inflammatory responses by secreting signaling molecules such as IL-10 and TGF-β, preventing non-physiological inflammation that could disrupt normal tissue patterning. Multiple lines of evidence indicate that macrophages serve as key mediators in the formation of new blood vessels. Studies on PU.1-deficient mice (which have severe defects in the myeloid lineage and lack skin-resident F4/80^+^ myeloid cells) show that macrophages are indispensable for the development of skin vasculature (8, 9). Macrophages secrete pro-angiogenic factors such as VEGF-A and PlGF to participate in the establishment of the dermal vascular network (38, 46).

As dermal structures mature, macrophages regulate fibroblast proliferation and extracellular matrix (ECM) synthesis, promoting the deposition of collagen and elastin fibers. This enhances the mechanical strength and functional integrity of the skin (47). This tissue remodeling and fibroblast-ECM regulatory program, established by macrophages during development, is critical. This program provides a blueprint for subsequent tissue repair; however, as we will discuss in Section 3.5, the aberrant reactivation and persistent activation of this very developmental program is also a core pathological mechanism in fibrotic diseases such as Systemic Sclerosis (SSc).During hair follicle development, macrophages maintain the cell density of the placode region by clearing excess epidermal cells, while simultaneously releasing TGF-β, IL-1, and Wnt-related factors that co-regulate follicle morphogenesis and inward growth (48). Additionally, studies suggest that macrophages in mouse skin may interact with the dermal papilla, facilitating the maturation of the lower follicular structures (49, 50).

After birth, the skin must rapidly establish barrier function and adapt to the external environment. Macrophages play a key role in maintaining local immune surveillance, regulating keratinocyte differentiation by recognizing microbial-associated molecular patterns (MAMPs). They are also quickly recruited to sites of mechanical injury, where they initiate repair programs. During skin formation, macrophages collaborate with other immune cells, including LCs and ILC2s. As antigen-presenting cells, macrophages promote the colonization of LCs in the epidermis. Furthermore, through the secretion of factors like IL-33, macrophages activate ILC2s to regulate hair follicle stem cell activity and contribute to the reformation of the epidermal barrier (51–53). In skin injury or inflammatory conditions, macrophages interact with neutrophils and myofibroblasts to enhance tissue repair (54, 55).

### 2.2 Langerhans cells

LCs are the only specialized antigen-presenting cells (APCs) in the epidermis of the skin, residing within the tightly interwoven layers of keratinocytes. LCs are a subtype of DCs derived from macrophage progenitors (11), distinguishing themselves from other DC subtypes by the expression of CD1a and CD207 (or langerin) (10). LCs possess characteristics of both macrophages and DCs, and have been recently described as “macrophages in DC disguise” (56). Unlike tissue-resident macrophages, LCs have the unique ability to migrate to regional lymph nodes. LCs are found in both murine and human epidermis, where they tightly associate with surrounding keratinocytes via homophilic interactions involving E-cadherin (also known as E-cadherin 1) (57).

Under steady-state conditions, most LCs develop from myeloid precursor cells. DC2-type cells and Gr-1^+^ monocytes can also differentiate into LCs within the skin (58, 59). In their resting state, LCs self-renew in the epidermis, maintaining homeostasis with the aid of keratinocytes and the TGF produced by LCs themselves. However, during inflammation, LCs are replenished by bone marrow-derived precursors (60, 61). Studies using murine models have demonstrated that LCs differentiation relies on several transcription factors associated with TGF-β signaling, including PU.1, RUNX3, and ID2, as well as interactions with interleukin-34 (IL-34) and the CSF1 receptor (MCSFR) (62–65). Notably, Tgfb2^-^/^-^ mice lack LCs (66). Interestingly, TGF-β1 signaling is also essential for maintaining the established LC network. Conditional knockout of *Tgfbr1*, *Tgfbr2*, or *Lamtor2* in LCs results in a loss of their ability to remain in the epidermis, causing them to migrate to regional lymph nodes (67).

*In vitro* studies indicate that human CD14^+^ monocytes, when cultured with GM-CSF, IL-4, and TGF-β, can differentiate into cells expressing Langerin (68). In contrast, CD1c^+^ dendritic cells, under the influence of GM-CSF, TGF-β, and BMP7, express higher levels of langerin, CD1a, and Birbeck granules than CD14^+^ monocytes (69).

**Figure 2:**
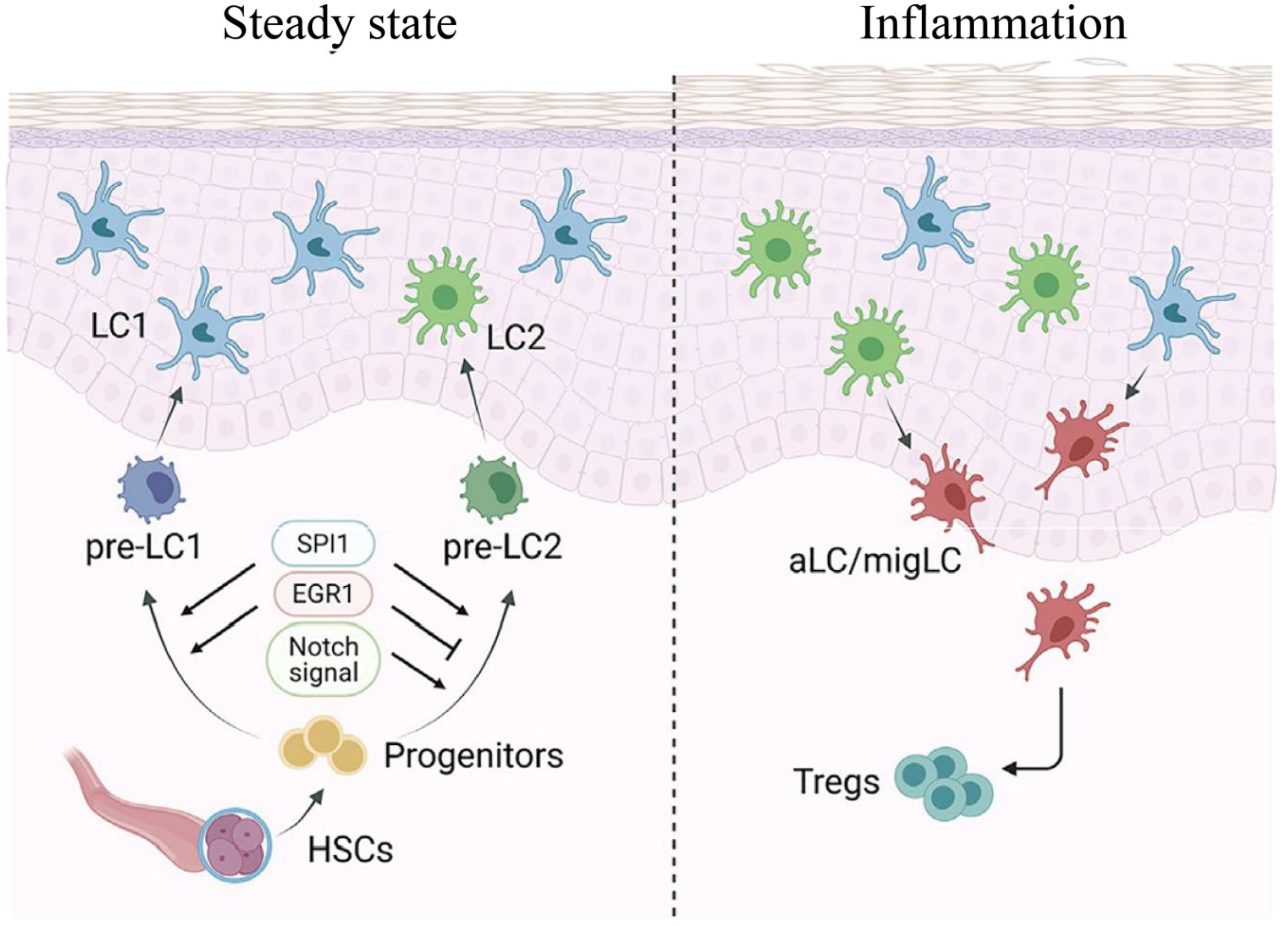
The full differentiation trajectories of LC derived from CD34+ hemopoietic stem cells of human cord blood and its regulation in inflammation. This figure illustrates the unique differentiation pathways exhibited by lymphoblasts (LCs) derived from CD34+ hematopoietic stem cells (HSCs) under different conditions. During steady state, precursor cells differentiate into two major subpopulations: LC1 and LC2. Under the regulation of SPI1, EGR1, and Notch signaling, they further differentiate into pre-LC1 and pre-LC2, ultimately maturing into the LC1 and LC2 subtypes in the epidermis. During inflammation, LC1 and LC2 activate to form aLC/migLC (activated/migratory Langerhans cells), interacting with regulatory T cells (Tregs) to participate in immune regulation within the inflammatory microenvironment. HSC, hematopoietic stem cell; LC, Langerhans cell; aLC/migLC, activated/migratory Langerhans cell; Treg, regulatory T cell. Copyright 2021, Elsevier Inc. Netherlands.

The primary function of LCs is to capture and process antigens from the skin and deliver them to T cells in the lymph nodes, thereby initiating adaptive immune responses (62). However, their exact role in the development of adaptive immunity and their functional heterogeneity remains difficult to fully define. Recently, Liu et al. (70) used single-cell RNA sequencing and mass cytometry to identify two steady-state LC subpopulations (LC1 and LC2) and two activated LC subpopulations in human epidermis and CD34^+^ hematopoietic stem cell-derived LCs (HSC-LCs) (**Figure 2**). LC1 is defined as the classical LCs, mainly involved in innate immunity and antigen processing. LC2 resembles monocytes or myeloid dendritic cells, participating in immune responses and leukocyte activation. EGR1 and Notch signaling pathways have been identified as key regulators of LC1 and LC2 differentiation. Notch signaling has also been shown to promote LC differentiation (71).

In the presence of GM-CSF, TGF-β1, and the Notch ligand DLL4, monocytes can differentiate into CD1a^+^ Langerin^+^ cells with Birbeck granules within 3 days. LCs possess immunosuppressive functions, capable of suppressing T cell responses induced by skin cDCs. For example, contact hypersensitivity induced by low doses of hapten applied percutaneously is enhanced in the absence of LCs (72). By inducing regulatory T (Treg) cells, LCs can protect mice from allergic contact dermatitis and experimental autoimmune encephalomyelitis (73).

### 2.3 Dendritic cells

In addition to epidermal LCs, the dermis also harbors DCs subsets with different origins, playing critical roles in antigen presentation, T cell recruitment, and tissue homeostasis maintenance. The common progenitors of macrophages/DCs differentiate into common DC precursors, which further give rise to various DC subsets, including CD141^+^ type 1 dendritic cells (cDC1s) (the murine homolog being XCR1^+^ cDCs), CD1c^+^ type 2 dendritic cells (cDC2s) (the murine homolog being CD11b^+^ cDCs), and CD123^+^ plasmacytoid dendritic cells (pDCs).

cDC1s specialize in cross-presenting antigens to CD8^+^ T cells and contribute to the induction of immune tolerance during the establishment of the neonatal skin barrier. Their differentiation is dependent on transcription factors such as Irf8, Irf4, Id2, and Batf3 (74, 75). In contrast, cDC2s are more efficient in guiding T cells towards Treg differentiation and help establish peripheral immune tolerance in the skin to maintain homeostasis. Their differentiation requires the expression of Id2 and Zeb2, in addition to IRF4 and Notch2/KLF4 (75–78). cDC2s represent a more abundant and heterogeneous subset. Villani et al. (79) identified two distinct populations within cDC2s in the blood and suggested that CLEC9a may serve as a better marker for recognizing cDC1 compared to CD141.

pDCs and cDCs are the only mature cell types that express the Flt3 ligand (Flt3L) receptor Flt3 (Flk2, CD135), which is otherwise expressed on multipotent and lymphoid progenitors (80, 81). Notably, Flt3L is not only necessary but also sufficient to support the exclusive development of pDCs and cDCs *in vitro* (82). pDCs are the major source of type I interferons, particularly IFN-α subtypes encoded by 1215 Ifna genes (83–85). Accordingly, pDCs are crucial for innate control of various viruses and/or T cell responses. Studies in neonatal mice have shown that pDCs rapidly accumulate in the skin after birth and activate interferon programs, aiding in the establishment of an “antiviral readiness state” that coordinates innate and adaptive immune responses (86, 87).

These DC subsets all originate from precursor cells in the bone marrow. Additionally, monocytes can also replenish the DC pool, particularly during inflammation, giving rise to monocyte-derived dendritic cells (moDCs) (88). These DCs, besides possessing monocyte characteristics, acquire transcriptional features similar to CD11b^+^ cDCs.

Overall, LCs and other dendritic cell subsets in the skin not only possess immune sensing and regulatory functions but also impact epidermal and dermal morphogenesis through intercellular interactions. This suggests that their roles in skin development extend far beyond traditional immune surveillance, with multiple layers of developmental regulatory potential.

### 2.4 Mast cells

MCs originate from CD34^+^ bone marrow precursors, but unlike other hematopoietic cells, they do not circulate in the bloodstream in their mature form (89). Instead, MCs precursors home to peripheral tissues, where they complete their differentiation under the influence of the local microenvironment. During this phase, they express high-affinity IgE receptors (FcϵRI) and the stem cell factor (SCF) receptor c-Kit, both of which are essential for their maturation, proliferation, and survival. MCs are located at key interfaces with the external environment, such as the respiratory tract, gastrointestinal tract, and skin. Based on the size of their secretory granules, MCs can be classified into two types: MCT (MCs that only release tryptase) and MCTC (MCs that also secrete chymase, carboxypeptidase, and tryptase-like proteases). MCT are found in mucosal tissues, such as the respiratory system and gastrointestinal tract, where they interact with external factors (e.g., food, pollen, drugs, microbes). In contrast, MCTC are located in the submucosal layer and connective tissue near the skin and conjunctiva (12, 13).

Most MCs in the skin are MCTC, predominantly located in the superficial dermis near blood vessels and nerves (90). MCs can be activated by immune signals, including antigen/IgE complexes, complement proteins, and Toll-like receptor (TLR) ligands, as well as by non-immune stimuli such as peptides from venom, calcium ionophores, and neuropeptides (91). Recently, members of the Mas-related G protein-coupled receptor (Mrgpr) family, such as *Mrgprb2* in mice and *MRGPRX2* in humans, have been identified as MC receptors (92–94). Both *Mrgprb2* in mice and *MRGPRX2* in humans are activated by basic secretagogues and are known to trigger MC degranulation via non-IgE mechanisms. The degranulation induced by these receptors exhibits distinct temporal and spatial dynamics compared to *FcϵRI*-mediated degranulation, with the release of cytoplasmic granules showing different patterns and characteristics in response to various activation stimuli (94, 95).

MCs not only participate in allergic responses but also play crucial roles in skin repair and wound healing. They can directly interact with DCs (96). IgE/antigen-activated MCs maintain long-term contact with DCs through lymphocyte function-associated antigen 1 (LFA-1) and very late antigen-4 integrins, causing cytoskeletal reorganization and leading to the release of MC granules and cytokines into DCs. This interaction allows IgE-bound antigens to transfer from MCs to DCs (96). In the skin, TNF secreted by MCs induces nearby dermal DCs to migrate to skin lymph nodes, thereby initiating T cell responses in models of allergic and irritant contact hypersensitivity (CHS) (97, 98). Similarly, DCs regulate the surface levels of TNF receptors on MCs, with TNF promoting DC maturation in a contact-dependent manner (90). Soluble mast cell-derived factors also contribute to the activation of Langerhans cell migration. In murine models, FcϵRI signaling in MCs drives the migration of LCs to skin-draining lymph nodes in a histamine-dependent manner (99).

### 2.5 Innate lymphoid cells

ILCs represent a previously underappreciated family of innate immune cells, whose discovery has prompted a paradigm shift in our understanding of tissue-resident innate immune cells and their roles in maintaining tissue homeostasis, as well as in inflammation induction, regulation, and resolution (8). The second group of ILCs (ILC2) was initially reported as cells that respond to IL-33 and IL-25 during helminth infection in adipose tissue and the small intestine, producing type 2 cytokines such as IL-5 and IL-13 (100, 101). It was subsequently found that ILC2 also exist in various organs, including the lungs, tonsils, liver, and muscles (102), and they are present in the skin as well (103). Key markers for defining ILC2 include the expression of the master transcription factor GATA3 and the ability to produce type 2 cytokines (e.g., IL-5 and IL-13). Flow cytometric analysis of specific ILC2 surface markers, such as IL-33R (ST2), is a more common method for their detection (104, 105). However, only a small portion of ILC2 in the skin express ST2 (106, 107). Single-cell RNA sequencing analysis of ILCs from the skin of IL-5 (Red5) reporter mice revealed that the expression of Icos, Ccr6, and Itgae was highly enriched in skin ILC2 (106). The highly complex and tissue-specific nature of ILC2 allows them to exhibit distinct response mechanisms to various stimuli. In healthy individuals, most of the effector skin ILCs are ILC2. Additionally, ILC3 have been found in the skin of both humans and mice with psoriasis (40, 108, 109), but the exact mechanisms of their function remain unclear.

In both the dermis and subcutaneous tissue, ILC2 subsets exhibit different characteristics (107). ILC2 expressing ST2, CD25, Sca-1, and KLRG1 (markers typically expressed on pulmonary ILC2) are enriched in the deepest layers of the skin, the subcutaneous tissue. ICOS^+^ CCR6^+^ ILCs are located in the epidermis and dermis, exhibiting ILC2 characteristics. While our understanding of ILC2 in mouse skin has advanced, very little is known about the localization and cell surface markers of ILC2 in human skin. Immunohistochemical analysis of ILC distribution in human skin shows that ILCs are present in the upper dermis of healthy skin, with the predominant subsets being ILC1 (T-bet^+^) and ILC3 (RORc^+^/AHR^+^). In atopic skin, the number of ILC2 increases (110).

Furthermore, other ILC subsets also play significant roles in skin development. ILC3 express the transcription factor RORγt and primarily secrete cytokines such as IL-17 and IL-22, which have diverse functions during skin development. After skin injury, activation of epidermal Notch1 signaling recruits ILC3 to the wound site. ILC3 rapidly produce IL-17F and IL-22, promoting macrophage recruitment and keratinocyte proliferation, thereby accelerating wound closure and reepithelialization (111). ILC3s and their secreted cytokines, IL-17 and IL-22, represent a double-edged sword within skin developmental and repair programs. As will be discussed in detail in Section 3.3, the IL-23-Th17 cell axis is pathologically hyperactivated in psoriasis, leading to an uncontrolled amplification of this repair program, which consequently drives the abnormal proliferation of keratinocytes and chronic inflammation. ILC3 also contribute to hair follicle regeneration (hair follicle neogenesis). They regulate macrophage and stem cell activity through secreted factors like CCL3 and influence the function of hair follicle stem cells by modulating BMP signaling (112). Additionally, in skin homeostasis, ILC3 form a network with keratinocytes, sebocytes, DCs, and macrophages to maintain barrier function and balance the commensal microbiota. They fine-tune epithelial immune responses through molecules such as IL-22 and TNF-α.

ILC1 depend on the transcription factor T-bet for development and mainly produce IFN-γ and TNF-α. They enhance the activation of macrophages and keratinocytes, thereby promoting skin barrier maturation and immune initiation (113, 114). Studies have shown that although ILC1 are sparse in the skin, they rapidly accumulate in inflammation models, such as contact dermatitis or psoriasiform dermatitis, and release IFN-γ, driving local Th1-type inflammation. This suggests an early immune regulatory role for ILC1 during wound or infection responses (115). For example, in the mouse imiquimod-induced dermatitis model, the number of ILC1 significantly increased between days 2-4, leading the early inflammatory response, outpacing NK cell accumulation (116). Moreover, ILC1 may play a role in angiogenesis and immune surveillance. The IFN-γ they release promotes macrophage phagocytosis and epithelial signaling, stimulating the expression of tight junction proteins, thus maintaining skin barrier function. However, research on the role of ILC1 in skin development is still limited and warrants further exploration.

## 3 Immune dysregulation and skin pathology: a development-pathology axis

Although immune cells are an indispensable part of normal skin development, any disruption in their function can lead to a variety of skin diseases, including congenital defects, autoimmune diseases, and chronic inflammation (**Table 2**). Traditionally, the development of skin immunity and its associated diseases have often been viewed as two separate biological contexts. However, mounting evidence compels us to break down this categorization and instead build a mechanistic bridge from development to disease. The pathophysiology of many chronic inflammatory skin diseases, such as AD and psoriasis, is largely not derived from *de novo* erroneous programming, but rather from skin cells entering a stable and abnormal transcriptional program (117).

**Table 2:** Immune cell dysregulation in pathological skin conditions.

Pathology	Key immune cells	Key mediators/Cytokines	Target cells	Key pathological outcome
Epidermolysis Bullosa (EB)	Neutrophils; Macrophages (M1-like);	TNF-α; IL-1β;	Keratinocytes; Wound site;	Excessive neutrophil infiltration; Chronic inflammation; Impaired wound healing; Blister formation.
Ichthyosis	Th17 cells; DCs; LCs;	Th17 pathway cytokines (e.g IL-17, IL-22)	Keratinocytes	Abnormal keratinization; Barrier dysfunction; Skin inflammation & scaling.
Atopic Dermatitis (AD)	Th2 cells; ILC2s;	IL-4; IL-13;	Keratinocytes; B cells; Sensory Neurons;	Barrier suppression (Filaggrin↓); IgE production; Intense Pruritus (Itch); Inflammation.
Psoriasis	Th17 cells; ILC3s; pDCs; Neutrophils;	IL-17A; IL-17F; IL-22; IFN-α;	Keratinocytes	Keratinocyte hyperproliferation (Acanthosis); Epidermal inflammation;
Systemic Lupus (SLE)	pDCs; double-negative 2 (DN2) B cells; Neutrophils (LDGs);	Type I IFN (IFN-α); NETs (DNA, histones); Autoantibodies;	All nucleated cells; pDCs; Platelets;	“IFN signature”; Inflammation; Immunothrombosis (vascular occlusion);
Systemic Sclerosis (SSc)	Macrophages (pro-fibrotic); Mast Cells;	TGF-β; IL-1β; CXCL8; CCL3; CXCL2; PTGS2	Fibroblasts (Myofibroblasts)	Excessive ECM deposition; Tissue remodeling; Fibrosis (skin hardening);

The critical question, however, is where this disease program originates, and growing evidence points toward developmental biology. This stable and abnormal transcriptional program represents a pathological reactivation of latent, highly conserved fetal developmental programs—a view strongly supported by recent single-cell atlas studies. These studies directly compared the cellular states of human fetal skin, healthy adult skin, and atopic dermatitis/psoriasis lesions, confirming that in inflammatory lesions, key cell populations like macrophages and vascular endothelial cells aberrantly re-exhibit transcriptional features active only during the fetal developmental stage (118).

Furthermore, critical developmental epigenetic regulatory mechanisms, such as genomic imprinting, have long been linked to susceptibility for atopic dermatitis and psoriasis (119), suggesting the importance of epigenetic states established during development for maintaining skin homeostasis in adulthood. Therefore, this section will explore the development-pathology axis, first focusing on the consequences of the failure of the developmental program itself, which leads to congenital diseases. Second, we will examine how the developmental origins of skin immunity lay the groundwork for future pathological states, and how these developmental programs are repurposed in disease to drive chronic inflammation and fibrosis.

### 3.1 Congenital skin diseases

Congenital skin diseases are typically caused by mutations in genes responsible for immune cell function, skin barrier formation, or tissue remodeling. These diseases may be associated with immune response deficiencies that disrupt normal skin development, resulting in fragile skin, deformities, and delayed wound healing.

Epidermolysis Bullosa (EB) is a rare and currently incurable genetic skin disease characterized by fragile skin and mucous membranes, with blister formation often triggered by minor trauma (120, 121). Although the main cause of EB is a defect in structural proteins, immune cells (such as macrophages and neutrophils) also play a role in the inflammatory response and blister formation. Especially in secondary infections or skin injuries, an overreaction of immune cells may lead to local inflammation, exacerbating the severity of the disease. Studies have shown that chronic wounds in EB patients reduce the proportion of macrophages in EB patients or EB mice, leading to the activation of pro-inflammatory cytokines like TNF-α and IL-1β, hindering wound healing (122, 123). Furthermore, macrophages in EB patients show changes in polarization profiles, with an increased prevalence of pro-inflammatory M1-like macrophages, resulting in chronic inflammation.

Neutrophils are the first professional phagocytes to reach the skin wound site, preventing infection by attacking any pathogens that invade the body through the open wound. However, this nonspecific attack can lead to tissue damage and chronic inflammation. Biopsy samples from skin with blisters in EB patients reveal elevated levels of chemokine receptors such as CXCR1, CXCR2, CCR2, and CCR4, along with dense infiltration of epidermal neutrophils (CXCR2+ CD11b+ CD16+), which make up the majority of the white blood cells (up to 90%) (124–126). This suggests that excessive neutrophil infiltration may be a key factor in the wound healing defects associated with EB.

In healthy development or acute repair, immune cells (such as macrophages) follow an orderly progression from a pro-inflammatory to a pro-remodeling phase. In EB, however, the persistent structural fragility resulting from incomplete structural development provides a non-stop damage signal. This prevents the immune system’s repair program from completing normally; macrophages are consequently stuck in a pro-inflammatory M1-like phenotype, while excessive neutrophil infiltration further exacerbates tissue damage. Therefore, structural developmental integrity is a prerequisite for the normal establishment and execution of immune repair and tolerance.

### 3.2 Autosomal dominant ichthyosis

Ichthyosis is a large and heterogeneous group of skin keratinization disorders. These conditions can be inherited or acquired, leading to defects in keratinocyte differentiation and abnormal epidermal barrier formation (127, 128). Regardless of the type of ichthyosis, many patients experience symptoms such as itching, recurrent infections, sweating disorders (hypohidrosis), poor heat tolerance, and various ocular, auditory, and nutritional complications, requiring regular monitoring. Traditionally, these diseases have been viewed as disorders of keratinocyte differentiation or mutations in structural proteins like filaggrin, loricrin, or transglutaminase-1.

DCs and LCs, as key antigen-presenting cells, reside in the epidermis or superficial dermis and participate in establishing local immune homeostasis immediately after birth. In LC-deficient mice, the barrier function of the stratum corneum is impaired, and transepidermal water loss increases significantly, suggesting the important role of these cells in keratinocyte maturation and barrier integrity (129). Recent studies on the immune characteristics of patients with four types of ichthyosis (Congenital Ichthyosiform Erythroderma (CIE), Lamellar Ichthyosis (LI), Epidermolytic Ichthyosis (EI), and Netherton Syndrome (NS)) have found that upregulation of the Th17 cell pathway is significantly associated with the severity of the disease. Activation of the Th17 pathway is thought to be a response to structural defects in the stratum corneum, leading to skin inflammation and scaling (130, 131). Additionally, in mice with abnormal numbers or function of Tregs, skin barrier dysfunction and abnormal epidermal keratinization occur, suggesting that Tregs may indirectly affect the keratinization process by modulating the Th17/Th2 axis (132).

Harlequin Ichthyosis (HI), a rare genetic disease caused by mutations in the ABCA12 gene, is the most severe form of autosomal recessive congenital ichthyosis. In HI skin, a deficiency in lipid transport due to ABCA12 gene mutation leads to increased skin permeability, triggering a range of clinical complications, notably hypernatremic dehydration, impaired thermoregulation, high risk of infection, nutritional difficulties and respiratory distress (133, 134). While HI is primarily a disorder of epidermal differentiation, studies show that immune responses are altered in these patients. The compromised skin barrier leads to increased susceptibility to infections, which in turn activates highly inflammatory immune responses. Immune cells, including DCs and T cells, participate in the chronic inflammatory response of HI, exacerbating skin lesions (130, 135). Growing evidence suggests that the occurrence of ichthyosis may not only be due to genetic defects in structural proteins but also involve abnormal activation or functional defects of immune cells during key windows of skin development.

### 3.3 Autoimmune and autoinflammatory diseases

Skin autoimmune and autoinflammatory diseases are typically characterized by abnormal immune responses, where self-tolerance is broken, and immune cells attack normal skin tissue. The molecular mechanisms behind these diseases usually involve T cells, B cells, DCs, and macrophages, which contribute to the destruction of skin structure.

Atopic Dermatitis (AD) is a highly prevalent chronic inflammatory skin disease that typically begins in early childhood and may subsequently progress to other allergic diseases such as asthma and allergic rhinitis. Its pathogenesis involves a complex interplay between genetic susceptibility, epidermal barrier dysfunction, and severe immune dysregulation. Although it was historically debated whether AD is primarily a barrier-defect-first or immune-defect-first disease, it is now widely accepted that these two factors are interconnected, forming a self-amplifying vicious cycle (136).

A core feature of AD is a compromised epidermal barrier, often associated with loss-of-function mutations in the filaggrin (FLG) gene. This barrier defect leads to increased transepidermal water loss and enhanced penetration of allergens, microbes, and irritants. In response to these external stimuli and mechanical scratching, epidermal keratinocytes release alarmins such as TSLP and IL-33, which are potent activators of the dominant “Type 2 immune response” in AD. This Type 2 response is rapidly initiated by ILC2s and sustained by adaptive Th2 cells. When triggered by factors like scratching or allergens, the acute phase begins, with Th2 cells proliferating and secreting IL-4 and IL-13. These cytokines drive B cell class-switching, leading to the production of large amounts of circulating Immunoglobulin E (IgE) and causing sensitization. More critically, IL-4 and IL-13 also act back on keratinocytes, further suppressing the expression of key barrier proteins like filaggrin and loricrin, thus perpetuating the barrier defect (136, 137).

Although acute AD lesions are clearly Th2-dominant, chronic AD lesions typically exhibit a mixed inflammatory infiltrate, with upregulation of Th1, Th17, and Th22 pathways. This phenomenon reflects the complexity and dynamic evolution of AD’s pathogenesis from its childhood onset through disease progression (138, 139). The study by Reynolds et al. found a significant increase in fetal-like macrophages (Mac2) and endothelial cells (VE3) in AD lesions. The conserved fetal genes in both cell types are enriched for “angiogenesis,” “leukocyte chemotaxis,” and “TGF-β signaling” pathways, which highly aligns with the pathological features of vascular abnormalities and immune infiltration in inflammatory skin diseases (118). More importantly, the number of these fetal-like cells significantly decreases as the clinical symptoms (EASI score) of AD patients improve following methotrexate treatment. This strongly suggests that these reactivated developmental programs are key drivers of disease activity, not mere bystanders.

Reynolds et al. also observed a significant expansion of fetal-like Mac2 and VE3 cells in psoriatic lesions. However, psoriasis has its own unique characteristics on the immune axis, with the activation of the IL-23-Th17 cell axis at its core (118). From the perspective of the development-pathology axis, the IL-17/IL-22 cell axis is precisely the developmental program described in Section 2.5 for normal skin wound repair and hair follicle regeneration. In psoriasis, this repair program becomes pathologically altered, leading to the uncontrolled proliferation of keratinocytes and chronic inflammation.

Psoriasis is a chronic inflammatory disease characterized by abnormal proliferation of keratinocytes and immune system dysregulation. Psoriasis includes several subtypes, including plaque psoriasis, guttate psoriasis, pustular psoriasis, and erythrodermic psoriasis. Among them, plaque psoriasis, also known as typical psoriasis, is the most common type, accounting for 80-90% of cases. It is characterized by well-defined erythema with silvery scales, often affecting the scalp, extensor surfaces, and lower back (140, 141). Immune system dysregulation is now considered the key driving factor in the pathophysiology of plaque psoriasis. Chronic inflammation in psoriasis is mainly caused by the presence of pathogenic T cell responses in the skin and pro-inflammatory feedforward signaling in keratinocytes (142–144). In mouse models such as experimental autoimmune encephalomyelitis, scientists have revealed the pivotal role of IL-23 in driving Th17 cell differentiation. These findings have been validated in skin lesion samples from human psoriasis patients. The IL-23–Th17 cell axis is central to psoriasis inflammation development, where IL-23 promotes the differentiation of T cells into IL-17-producing CD4+ T helper cells (Th17), CD8+ cytotoxic T cells (Tc17), and IL-22-producing CD4+ T helper cells (Th22). IL-23 also stimulates T cells to produce IL-17A, IL-17F, and IL-22, which are key effector cytokines in the pathogenesis of psoriasis, activating and proliferating keratinocytes (144–146). MCs, γδT cells, αβT cells, and ILCs also produce IL-17A, and most of these depend on IL-23 (145, 147–149). Numerous neutrophils are found in the upper epidermis of psoriatic plaques; while they do not produce IL-17A, they capture and release IL-17A from extracellular traps, a core defense and inflammatory function of neutrophils (150) In addition to IL-23, the differentiation of naive T cells into Th17 and Tc17 cells may also be influenced by other cytokines. Inflammatory macrophages and myeloid dendritic cells have been shown to produce IL-1, IL-6, and transforming growth factor-β (TGF-β), all of which drive Th17–Tc17 polarization (151).

Type I interferon signaling plays a key pathogenic role in psoriasis. Studies have found that systemic treatment with IFN-α and topical treatment with imiquimod (which induces local IFN-α production) both stimulate the development of human psoriasis (152, 153), while inhibition of this signaling pathway can lead to clinical improvement in psoriasis patients (154, 155). Apart from keratinocytes, IFN-I is primarily produced by plasmacytoid dendritic cells (pDCs). During the early stages of psoriasis, nucleic acid fragments released by damaged keratinocytes can bind to antimicrobial peptides (e.g., LL37), forming immune-stimulatory complexes that activate TLR7 and TLR9 pathways in pDCs, triggering the production of large amounts of IFN-α (156). These interferons bind to surface receptors IFNAR1/2, activate JAK1/TYK2, and induce STAT1/STAT2 phosphorylation, forming the ISGF3 complex with IRF9, which drives the expression of various interferon-stimulated genes (ISGs), further amplifying inflammatory signals (157). IFN signaling can activate the IL-23–Th17 cell axis. IFN-I strongly promotes the maturation and activation of DCs, significantly upregulating the expression of their MHCII, CD80/CD86, and pro-inflammatory cytokines such as IL-12 and IL-23, inducing T cells to polarize towards Th1 and Th17. LGALS9 secreted by DCs is received by CD44+ dermal fibroblasts, promoting the expression of extracellular matrix (158). Furthermore, IFN-I can activate CD8+ T cells, inducing them to produce IL-17A and IFN-γ, forming Tc17 and Tc1 cells (159). These cells infiltrate psoriatic plaques, exacerbating epidermal damage and inflammatory responses.

Recently, increasing evidence has shown that ILCs play a role in various pathologies. ILCs are also major sources of Th17 cytokines such as TNF-α, IL-17, and IL-22, indicating that ILCs and Th17 cells work together through innate immune responses in psoriasis. A significant increase in NKp44+ ILC3 cells was found in the skin and blood of psoriasis patients, and this increase was associated with the severity of psoriasis (40, 108). In addition, mature ILC subgroups can alter their phenotype and function according to local environmental factors, exhibiting plasticity similar to Th cells. NCR-negative ILC3 can be converted to NCR+ ILC3 by co-culturing with IL-1β and IL-23, both of which have been found to be elevated in psoriatic lesions and are related to the pathogenesis of psoriasis (101). Although classic ILCs express CD127, Velandia et al. (160) identified a non-classic ILC subgroup in peripheral blood samples from healthy donors and psoriasis skin biopsies, Lin- CD123+ CD127low cells, which also expressed IL-22 and IL-17, contributing to the immunopathological features of psoriasis.

**Figure 3:**
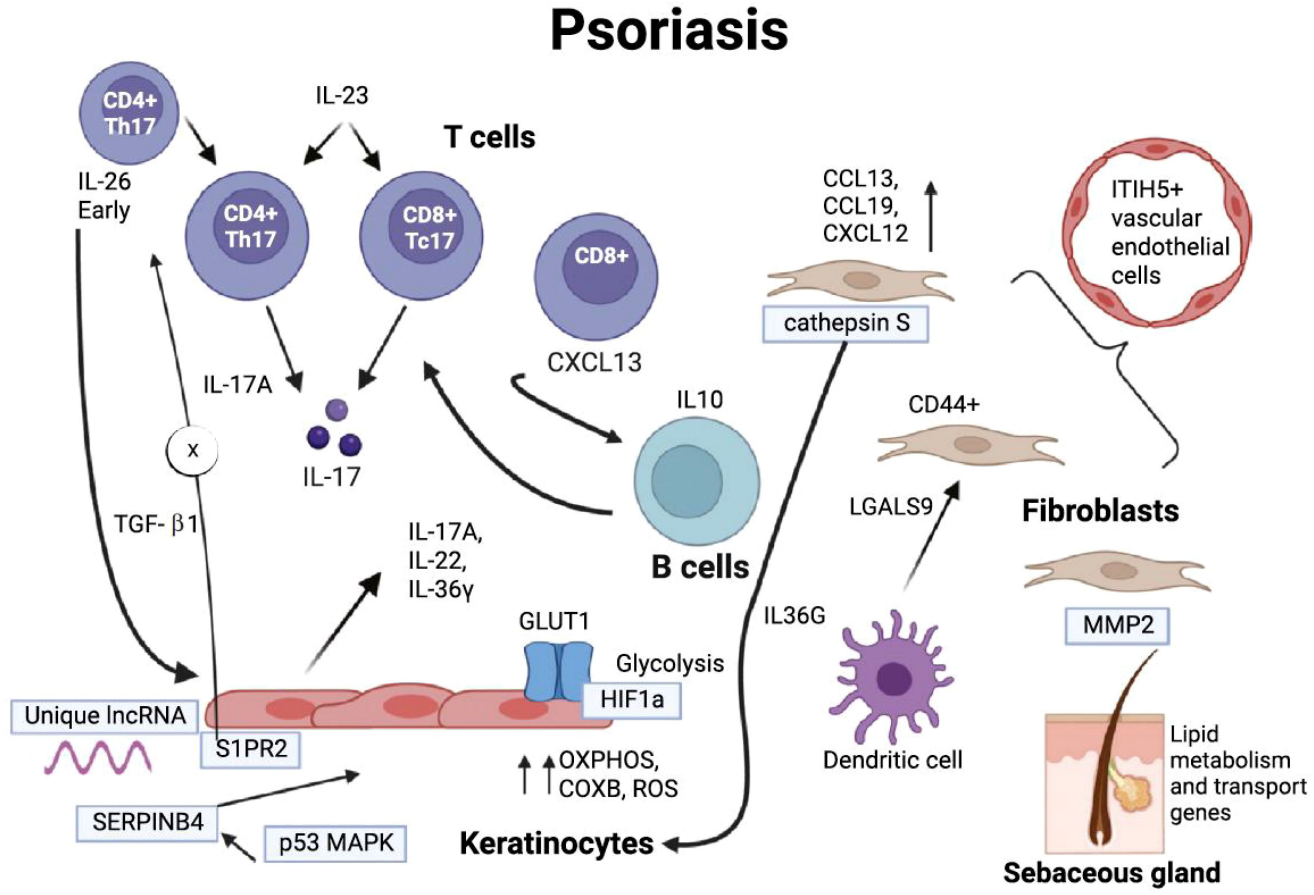
Insights from ST studies into the pathophysiology of psoriasis. Basal epidermal cells exhibit high competition for glucose, using glucose metabolism and the production of reactive oxygen species (ROS) to promote the upregulation of OXPHOS and COX, maintaining excessive keratinocyte proliferation. Additionally, HIF-1α acts on GLUT1, further promoting epithelial remodeling. The expression of S1PR2 inhibits the recruitment of Th17 cells to the skin. Keratinocytes contain a large number of differentially expressed and specific lncRNAs, which are significantly related to cell proliferation and epidermal differentiation. PSO-related autoantigen SERPINB4 activates the p38 MAPK that contributes to keratinocyte inflammatory responses. OXPHOS, oxidative phosphorylation; ROS, reactive oxygen species; MAPK, mitogen-activated protein kinase; GLUT1, glucose transporter type 1. Copyright 2025, Elsevier Inc. Netherlands.

The development of spatial transcriptomics (ST) technology has enhanced our understanding of the cellular and genetic characteristics in psoriasis. Leung et al. (161) summarized the use of ST combined with single-cell RNA sequencing (scRNA-seq) to analyze the tissue microenvironment and cellular interactions in psoriasis (**Figure 3**).

### 3.4 Systemic lupus erythematosus

Systemic lupus erythematosus (SLE) is a prototypic systemic autoimmune disease characterized by the persistent production of autoantibodies targeting diverse intracellular and cell-surface antigens, including antinuclear antibodies (ANA), anti-double-stranded DNA (anti-dsDNA), anti-Smith (Sm), anti-ribonucleoprotein (RNP), and antibodies to Sjögren’s syndrome-related antigens A (SSA/Ro) and B (SSB/La) (162, 163). Plasmablasts are the predominant source of anti-dsDNA autoantibodies, whereas long-lived plasma cells largely produce autoantibodies against extractable nuclear antigens such as Sm and RNP (147, 148). The spliceosome complex containing U1 small nuclear RNA (U1-RNA) and its associated Sm and RNP proteins is a major immunological target in SLE and may provide mechanistic insight into the molecular drivers of autoimmunity in this disease (164).

**Figure 4:**
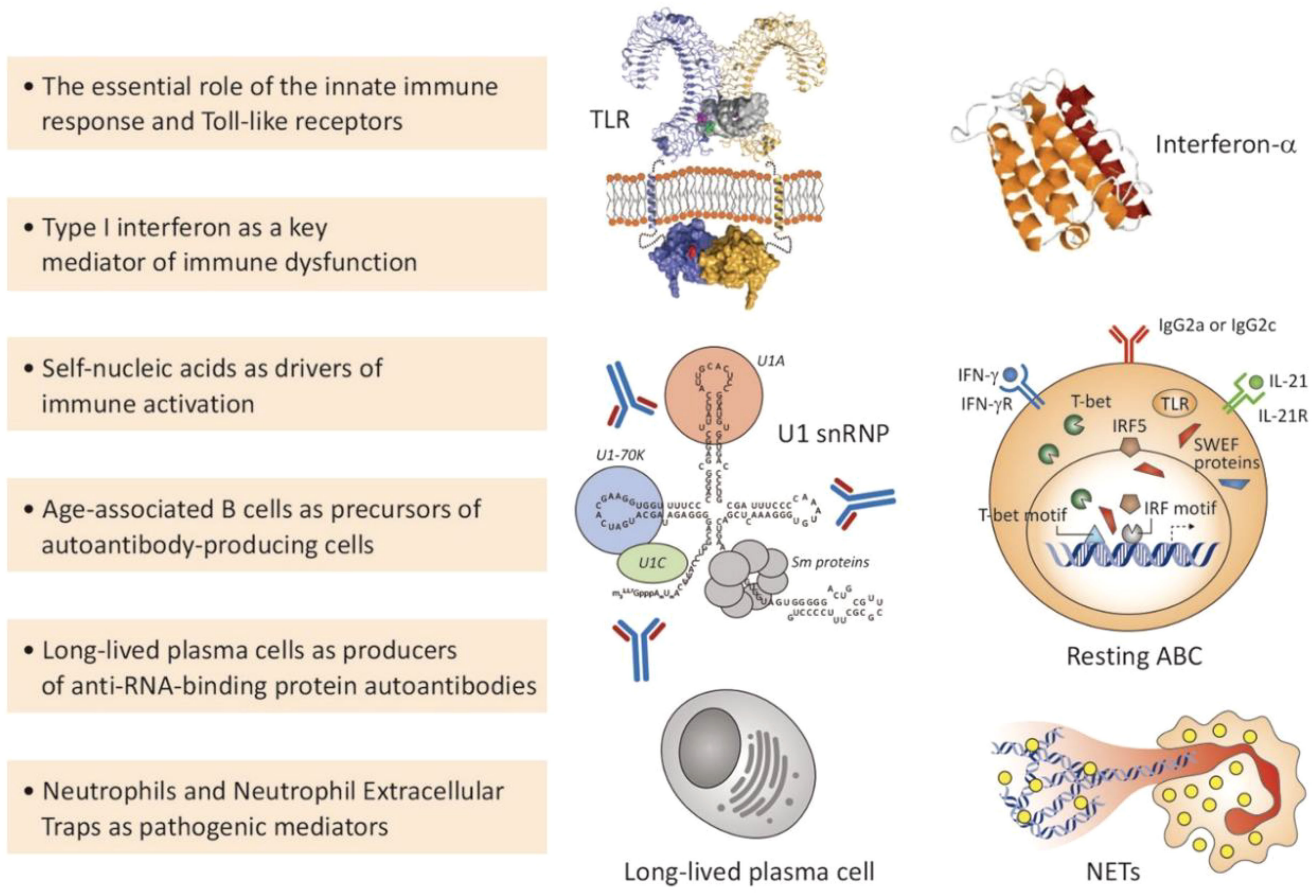
Selected key scientific advances relevant to SLE pathogenesis. This figure highlights six core pathogenic mechanisms and cellular participants in SLE, including: (1) the pivotal role of innate immune responses, particularly recognition of self-nucleic acids by endosomal Toll-like receptors (TLRs); (2) type I interferon (IFN-α) as a central mediator of systemic immune dysfunction; (3) the role of endogenous nucleic acids (e.g., U1 small nuclear ribonucleoprotein complexes) as drivers of immune activation; (4) the emergence of auto-reactive age-related B cells (ABCs) driven by factors such as T-bet and IRF5; (5) the persistent production of autoantibodies by longevity plasma cells; (6) the formation of neutrophil extracellular traps (NETs) by neutrophils, releasing potent autoantigens and amplifying inflammatory responses. TLR, Toll-like receptor; IFN, interferon; snRNP, small nuclear ribonucleoprotein; ABC, age-related B cell; NETs, neutrophil extracellular traps. Copyright 2023, Elsevier Inc. Netherlands.

The skin is a frequent target organ, with 75–80% of patients developing cutaneous manifestations (165–168). The most characteristic lesion is the malar or “butterfly” rash, localized to the cheeks and nasal bridge, resulting from inflammation at the dermal–epidermal junction. SLE pathogenesis is multifactorial, shaped by genetic susceptibility, immune dysregulation, environmental triggers, and hormonal influences (**Figure 4**). Among these, endosomal nucleic acid-sensing Toll-like receptors (TLRs) are central molecular hubs, integrating signals from aberrantly activated immune cells—including DCs, B cells, T cells, neutrophils, and macrophages—to drive disease initiation and perpetuation.

pDCs are key sentinels in SLE, activated in response to endogenous nucleic acids released during ultraviolet light exposure, viral infection, or defective apoptotic cell clearance. These nucleic acid–immune complexes engage TLR7 and TLR9, triggering robust type I interferon (IFN-α) production (169, 170). The resulting interferon signature—characterized by upregulated interferon-stimulated genes (ISGs) in keratinocytes and other stromal cells—facilitates conventional DC maturation, enhances antigen presentation, promotes B cell activation, and skews T cell differentiation toward Th1 and Th17 phenotypes. This creates a self-sustaining inflammatory milieu tightly correlated with clinical disease activity. In normal skin development, pDCs help establish an antiviral readiness state and discriminate between self and non-self. In SLE, however, pDCs erroneously recognize self-nucleic acids released from apoptotic cells as a threat—a departure from the developmental program of peripheral immune tolerance—and subsequently drive autoimmune responses in B and T cells.

B cell tolerance checkpoints are impaired in SLE, enabling autoreactive clones to persist in the periphery, which contradicts the mechanisms of central and peripheral immune tolerance. Recently identified age-associated B cells (ABCs)—defined by a CD11b^+^ CD11c^+^ T-bet^+^ phenotype—are expanded in murine lupus models and in SLE patients (171–175). Their human counterpart, double-negative 2 (DN2) B cells (IgD^-^ CD27^-^ CXCR5^-^ CD11c^+^ T-bet^+^), is enriched in patients with active disease and lupus nephritis (176, 177). ABC/DN2 cells are highly responsive to TLR7 signaling and rapidly differentiate into autoantibody-secreting plasma cells. T follicular helper (Tfh) cells, which are also increased in SLE, provide potent B cell help via ICOS and CD40L, supporting germinal center reactions, affinity maturation, and class-switch recombination, ultimately producing high-affinity pathogenic autoantibodies (178, 179). Immune complex deposition in target tissues such as glomeruli and skin triggers complement activation, leukocyte recruitment, and local tissue injury.

Effector T cell subsets are imbalanced, with Th1 and Th17 cells producing proinflammatory cytokines (IFN-γ and IL-17A, respectively) that activate macrophages, neutrophils, and endothelial cells, sustaining chronic inflammation. Tregs are numerically reduced or functionally impaired, weakening suppression of autoreactive responses (180–183). Abnormal expansion of Tfh cells not only amplifies B cell activation but also undermines regulatory pathways, serving as a central nexus of T–B cell crosstalk dysregulation.

Neutrophils, particularly a pro-inflammatory subset known as low-density granulocytes (LDGs) which are expanded in SLE, exhibit an enhanced propensity for NETosis. This process is now clearly defined as a distinct form of programmed neutrophil cell death, characterized by the decondensation and extrusion of chromatin (DNA and histones) complexed with granular proteins such as myeloperoxidase, neutrophil elastase to form neutrophil extracellular traps (NETs). These NETs are potent drivers of the pathogenic type I interferon (IFN-I) signature in SLE. This mechanism involves two primary arms: First, the DNA-rich NETs, often complexed with autoantibodies or antimicrobial peptides like LL37, are internalized by plasmacytoid dendritic cells (pDCs) and engage endosomal TLR9, a key sensor that triggers robust IFN-I production. Second, NET-derived DNA that accesses the cytosol of other cells, such as monocytes and macrophages, activates the cGAS–STING pathway, which further amplifies the systemic IFN-I response (184–188). Furthermore, interactions between platelets and neutrophils significantly exacerbate this pathway. Within the inflammatory environment of systemic lupus erythematosus, activated platelets bind to neutrophils via mechanisms such as P-selectin binding to PSGL-1, releasing mediators from activated neutrophils that substantially lower the threshold for NETosis initiation (189). This interaction creates a vicious cycle. Released NETs act as potent prothrombotic scaffolds, promoting platelet aggregation and fibrin deposition through dual mechanisms of “physical scaffolding” and “chemical stimulation,” thereby linking thrombosis and inflammation in SLE (190).

Macrophages and monocytes display defective clearance of apoptotic debris (191, 192), resulting in persistent autoantigen exposure. Type I interferon primes these cells toward a proinflammatory phenotype, enhancing antigen presentation but impairing phagocytic capacity (193–195). In inflamed tissues, macrophages secrete IL-6, TNF-α, and IL-1β, amplifying local inflammation and sustaining autoreactive T and B cell responses (193, 196). Monocytes in SLE exhibit transcriptomic reprogramming, including upregulated TCN2 expression, which augments TLR4-mediated inflammatory responses and tissue injury (197, 198).

NK cells are numerically and functionally compromised in SLE (199–201). Increased CD38 expression and defective upregulation of SLAMF1/SLAMF7 impair NK-mediated clearance of autoreactive immune cells, potentially facilitating the persistence of long-lived autoantibody-secreting plasma cells (202–206).

ILCs have emerged as additional players in SLE immunopathology. Increased ILC1 and ILC3 frequencies, alongside reduced circulating ILC2, have been reported in SLE patients (207). Some studies identify ILC1 as the dominant circulating subset, decreasing after immunosuppressive therapy (208), while others report no significant changes (209). Such discrepancies may reflect disease heterogeneity, treatment effects, or methodological differences. Defining the mechanistic contribution of ILC subsets to SLE pathogenesis may reveal novel therapeutic targets.

### 3.5 Systemic sclerosis

Systemic sclerosis (SSc), also known as scleroderma, is a connective tissue disorder characterized by fibrosis of the skin and internal organs, vascular damage, and immune dysregulation (210). Skin changes, including thickening, hardening, and tightening, are primarily due to excessive extracellular matrix (ECM) deposition. Extensive research on inflammatory cells and their mediators has shown that, in addition to fibroblasts, macrophages play a crucial role in the initiation and progression of SSc fibrosis. The synergistic interaction between fibroblasts and macrophages can influence their cellular responses, increasing tissue thickness and stiffness (211), thereby coordinating the onset or resolution of fibrosis within tissues (212–215).

Beyond the classical paradigm of macrophage polarization, new macrophage subsets have been identified in relation to tissue repair and fibrosis remodeling (216, 217). For example, distinct macrophage phenotypes (LYVE1^lo^ MHC-II^hi^ and LYVE1^hi^ MHC-II^lo^) exhibit specific niche-dependent functional roles in pulmonary and cardiac fibrosis. Li et al. (218) conducted single-cell RNA sequencing (scRNA-seq) clustering analysis of macrophages in skin biopsies from SSc patients and identified four macrophage clusters. One cluster, identified as pro-inflammatory macrophages, not only expressed high levels of pro-inflammatory cytokines such as IL-1β, CXCL8, CCL3, CXCL2, and PTGS2, but also upregulated NR4A1, NR4A2, and NR4A3, which are direct early response genes to various stimuli and are implicated in the pathogenesis of fibrosis tissue remodeling.

There is extensive intercellular communication between fibroblasts and macrophages, with 393 significant interactions identified. These interactions may facilitate the transition from homeostasis to a fibrotic state. In SSc patients, the number of monocytes in peripheral blood is significantly increased, with a reduced apoptosis rate, further promoting their differentiation into macrophages (219–221). In early SSc lesions and unaffected skin, mast cell proliferation and activation occur prominently, although degranulated MCs are only elevated in the affected skin. MCs can degranulate in allergic responses under the influence of IgE, releasing a mixture of cytokines, growth factors, and proteoglycans (222). Among these, transforming growth factor-β (TGF-β) is a key driver of SSc fibrosis (223), and MCs are a major source of TGF-β in SSc patients (224).

As described in Section 2.1, macrophages construct the dermis during embryonic development by regulating fibroblast proliferation and ECM synthesis. In SSc, the interaction between them is pathologically activated. Macrophages persistently release the pro-fibrotic factor TGF-β, causing fibroblasts to continuously deposit ECM, which ultimately leads to skin hardening.

## 4 Emerging therapeutic directions and future prospects

The understanding of immune cells in skin development and disease has opened new avenues for therapeutic interventions aimed at modulating immune responses to restore skin homeostasis. Immunomodulatory therapies offer promising strategies for treating skin diseases related to immune dysregulation, such as congenital skin diseases, autoimmune disorders, and fibrotic diseases. Traditional treatments often suffer from issues like strong side effects, high toxicity, poor targeting, and low treatment efficiency. With the continuous advancement of precision medicine, the design of immunomodulatory therapies tailored to individual patients based on their genetic and immune profiles has become possible. In this section, we explore the emerging therapeutic methods, the challenges associated with targeting immune cells, and the potential future directions for improving skin regeneration and immunomodulation in clinical settings.

Gene therapy provides a promising approach to address developmental skin diseases caused by potential genetic mutations. This includes the use of viral and non-viral vectors combined with scaffolds, RNA interference, and CRISPR-based gene editing. For example, in severe blistering diseases such as epidermolysis bullosa, gene therapy is being explored as a method to correct genetic defects in skin cells, potentially improving skin integrity and reducing blister formation. Pathogenic COL7A1 gene (C7) mutations are the primary cause of recessive dystrophic epidermolysis bullosa (RDEB), and restoring the normal expression of COL7A1 and producing type VII collagen is critical for treating RDEB. Various viral vectors have been developed to deliver COL7A1, including lentivirus and herpes simplex virus 1 (HSV-1) vectors. Gurevich et al. (225) developed a vector called beremagene geperpavec (B-VEC), which restores type VII collagen synthesis in the keratinocytes and fibroblasts of RDEB patients. B-VEC has been approved by the U.S. Food and Drug Administration (FDA) and recently recommended for approval by the European Medicines Agency (EMA). Lipid and biopolymer nanoparticles are promising alternatives to viral nucleic acid packaging and delivery. Zeng et al. (226, 227) utilized high-branched poly (β-amino ester) (HPAE) to encapsulate circular DNA containing the COL7A1 transgene, which was injected intradermally in mice, effectively restoring type VII collagen in RDEB keratinocytes. Non-viral vectors, including liposomes, hydrogels, microneedles, peptides, and metal nanoparticles, are used in various disease types and personalized approaches. Wang et al. (228) explored hyaluronic acid-based microneedles to deliver polymer nanoparticles containing dexamethasone for treating inflammatory skin diseases. Chen et al. (229) used SPACE peptides for the local delivery of siRNA in treating skin diseases.

CRISPR/Cas9 technology has shown tremendous promise in gene correction due to its simple, inexpensive, and universal editing mechanism. The homology-directed repair (HDR) method mediated by this technology can correct mutations in exons and restore C7 protein expression (230–232). However, the HDR efficiency of CRISPR/Cas9-mediated gene editing remains low. Recently, a programmable single-base editing (BE) method has been developed to precisely and irreversibly deaminate one nucleotide to another without the use of DNA double-strand breaks and HDR (233–235).

Mesenchymal stem cell (MSC)-derived extracellular vesicles (EVs) have shown great potential in skin repair and regeneration. MSCs are known for their immunomodulatory properties, capable of influencing immune cell activity and creating specific immune microenvironments that help reduce inflammation, scarring, and support tissue regeneration. Some studies suggest that paracrine interactions between MSCs and neighboring cells (such as the secretion of bioactive substances like cytokines, chemokines, and extracellular vesicles) lead to healing effects, rather than cell transplantation. EVs are nano-sized particles actively secreted by cells and play important roles in cellular communication, genetic material transfer, signal pathway regulation, and nutrient management (236–239). MSC-derived EVs can stably transport cargo, offering advantages such as low immunogenicity, low tumorigenicity, low cytotoxicity, ease of storage, high stability, low degradability, ability to penetrate the blood-brain barrier, and potential for intravenous injection. These vesicles can also be internalized into cells and have inherent homing properties. For instance, Zeng et al. (240) utilized subcutaneous injections of human adipose-derived MSC exosomes to reduce inflammatory cytokine levels and alleviate atopic dermatitis-like symptoms. MSC-derived miRNAs participate in multiple processes of wound healing, including regulating inflammation, promoting angiogenesis, and regulating cell proliferation, migration, and apoptosis (241, 242).

Advanced technologies, such as spatial transcriptomics (ST) and scRNA-seq, have provided researchers with unprecedented tools to dissect the complex interactions between immune cells and skin tissues in both normal development and disease states) (243, 244). For example, studies using these methods have found significant differences in the organization of immune cells and their gene expression patterns in the skin of psoriasis patients compared to healthy individuals. Jiang et al. (158) used scRNA-seq, ST, and immunostaining to discover that DC-secreted LGALS9 signals received by CD44+ dermal fibroblasts lead to an increase in extracellular matrix expression, hardening the dermal environment and thickening/hardening psoriatic lesions. Inhibiting the LGALS9-CD44 pathway alleviated psoriasis symptoms. Mitamura et al. (245) combined ST and scRNA-seq to identify unique fibroblasts, DCs, and macrophage clusters in atopic dermatitis, revealing unknown cellular crosstalk in leukocyte-infiltrated areas of the lesions.

Fibrotic skin diseases (such as systemic sclerosis, hypertrophic scars, and psoriasis) are characterized by extensive immune cell infiltration, which may affect the development of fibrotic lesions by releasing inflammatory mediators and regulating extracellular matrix synthesis. Deng et al. (246) used fluorescence-activated cell sorting to isolate CD45+ cells from keloids and performed scRNA sequencing, finding an increase in Th17 cells and investigating Th17-fibroblast interactions. Recently, extensive analyses of pro-fibrotic cells in mouse skin wound healing, fibrosis, and aging processes revealed a macrophage cluster expressing galactose-type C-type lectin 2 (Mgl2/CD301b). This macrophage subset promotes skin repair by enhancing proliferation and fibroblast re-proliferation (55). Shook et al. (247) showed that transplantation of CD206+/CD301b+ macrophages into mouse wounds significantly promoted skin repair. Our team has developed biomimetic nanocapsules based on CD301b+ macrophage revitalization, significantly improving the therapeutic effect on inflammatory bone diseases (248, 249). ST and scRNA-seq have enabled researchers to identify new cell populations, deepen understanding of known cell types, and study various fundamental biological processes, including cell differentiation, lineage tracing, and developmental trajectories, which are reflected across different organisms, including skin development. In particular, spatial transcriptomics has proven invaluable in illustrating complex communication pathways between different cell types (including pathogenic cells), especially in various skin diseases. The detailed molecular information obtained through these technologies holds tremendous potential for improving diagnostic methods and developing more targeted and effective therapeutic strategies for inflammatory skin diseases.

The development of tissue-engineered skin using 3D bioprinting represents a cutting-edge approach in the field of skin wound healing and grafting. Full-thickness skin replacement is limited by the shortage of donor skin, immune rejection, and the risk of infection (250) 3D bioprinting is considered to have a profound impact on the field of tissue engineering. Over the past decades, a variety of 3D-bioprinted skin models have been developed for applications such as skin irritation testing and drug efficacy screening (251). Three widely applied 3D bioprinting techniques include extrusion-based bioprinting (EBB), laser-assisted bioprinting (LAB), and droplet-based bioprinting (DBB) (**Table 3**).

**Table 3:** 3D biological printing methods.

Methods	Components	Advantages	Challenges	Ref.
EBB	Automated machine systems Fluid distribution systems	Wide range of printable bioink types Print porous grid structures	Low resolution	(252–256)
LAB	Pulsed laser source Laser focusing tool Laser energy-absorbing metal ribbon film Receiving substrate	Print non-contact High cell activity Printing with high resolution	Lack of an appropriate fast gelation mechanism	(257, 258)
DBB	Inkjet bioprinting (IJB) Electrohydrodynamic (EHD)	Higher precision Minimal waste of bioink	Porous structure cannot be produced Inkjet aperture is easily blocked	(259, 260)

While skin constructs hold great promise for wound healing and regeneration, their structural integrity remains limited due to the absence of skin appendages—critical components essential for the true functional restoration of skin. To address this limitation, various approaches have been developed to print complex skin structures, including hair follicles, vascular networks, and full-thickness skin models (**Table 4**). For example, Catarino et al. (263) utilized 3D bioprinting to automate the reconstruction of complex human hair follicle models. They created a concentric multilayered cellular structure that mimics the three-dimensional organization of human hair follicles. This model serves as a robust high-throughput platform for assessing the potential toxicity or regenerative capacity of substances on follicular cells. The approach not only enables large-scale production of isolated spheroids but also supports the fabrication of reconstructed skin models containing hair follicles with higher resolution, speed, and flexibility. However, the follicles in this model still lack the advanced differentiation of epidermal cells surrounding the dermal papilla (268, 269).

**Table 4:** The structure of 3D bioprinting of skin.

Classifications	Materials	Cell (origin)	Advantages	Ref.
Hair	Gelatin methacrylate (GelMA) Hyaluronic acid methacrylate (HAMA)	Hair follicle dermal papilla cells (HFDPC) Normal human dermal fibroblasts (NHDF)	Micron-precision multi-layer skin substitutes Spontaneously form pore structures	(261)
	Magnesium silicate (MS) nanospheres	Normal human dermal fibroblasts Dermal papilla cells	Good follicular formation and angiogenesis Promote skin regeneration	(262)
	BioX bioprinter	Dermal papilla cells (DPCs) Human umbilical vein cells (HUVECs)	Automated reconstruction of complex human hair follicle models	(263)
Vascularization	strontium silicate (SS) microcylinders	Fibroblasts Blood vessel endothelial cells	Support cell proliferation and angiogenesis	(264)
	GelMA HAMA	Epidermal stem cells (Epi-SCs) Skin-derived precursors (SKPs)	Scarless healing Regenerating hair follicles, blood vessels, sebaceous glands and other skin appendages	(265)
	Tetrameric self-assembling peptides IVFK (Ac-Ile-Val-Phe-Lys-NH2) and IVZK (Ac-Ile-Val-Cha-Lys-NH2)	Human dermal fibroblasts Epidermal keratinocytes	Significantly promotes the proliferation of epidermal cells Activate autocrine and paracrine signaling	(266)
	polyglycolic acid (PGA) Xeno-free dermal Epidermal bioink	Human endothelial colony forming cells (HECFCs) FBs (human dermis) PCs (human placentas) KCs (human epidermis)	A xeno-free approach to complex tissue engineering was achieved.	(267)

Ma et al. (265) proposed a prefabricated, cell-laden artificial skin strategy for skin repair. Using a photosensitive multi-component hydrogel (5% GelMA–0.5% HAMA) as the biomaterial, combined with epidermal stem cells (Epi-SCs) and skin-derived precursor cells (SKPs), they fabricated artificial skin capable of achieving complete regeneration of full-thickness skin wounds in nude mice. The regenerated tissue displayed epidermis, dermis, vasculature, capillaries, and sebaceous glands closely resembling those of healthy skin.

At present, research on creating vascularized skin structures containing functional skin appendages remains limited. Moreover, most current medical applications of 3D bioprinting are restricted to non-living structures and have not yet reached clinical implementation. Future studies should focus on developing novel strategies, coupled with more innovative research and clinical trials, to advance the application of 3D bioprinting in skin regeneration.

## 5 Conclusion

The development and maintenance of the skin are critically dependent on a precisely orchestrated interplay of various cell types, with immune cells emerging as key regulators throughout this complex process (1, 8–11). This review has highlighted the significant contributions of macrophages, DCs, MCs, regulatory T cells, and innate ILCs to fundamental aspects of skin development. Disruptions in this delicate immunological control can lead to a spectrum of developmental pathologies. The purpose of this review is to move beyond isolated descriptions of development and disease and instead establish a development-pathology axis. Congenital disorders, often stemming from genetic defects affecting the immune system, Congenital diseases such as EB and NS provide the proof for this axis. They demonstrate how a single-point failure in structural developmental programs inevitably leads to the dysregulation of immune repair programs. can manifest with a variety of skin abnormalities, including increased susceptibility to infections and chronic inflammation (4, 22–25). Autoimmune diseases arise from a misdirected immune response against the skin’s own tissues, leading to conditions like psoriasis, vitiligo, and scleroderma. The pathological mechanisms of AD, psoriasis, SLE, and SSc can, to a large extent, all be re-interpreted as the pathological activation of normal fetal developmental programs. Aberrant immune responses can also contribute to the development of fibrotic skin diseases, characterized by the excessive deposition of extracellular matrix.

The growing understanding of these immunological mechanisms is driving the development of novel therapeutic strategies. Immunomodulatory approaches, including stem cell therapies and gene therapy, hold significant promise for promoting skin regeneration and treating developmental pathologies. Furthermore, the advent of advanced technologies like spatial transcriptomics and single-cell sequencing is revolutionizing our ability to study the intricate crosstalk between immune cells and skin tissues at high resolution (236–240), providing unprecedented insights into the molecular underpinnings of both normal skin development and disease.

Future research should focus on further elucidating the precise molecular mechanisms that mediate the interactions between immune cells and other skin cells during different stages of development. We should not be satisfied with merely describing which cytokines are elevated in disease, but must answer a more fundamental question: Why is this pathway, which is crucial for development and repair, abnormally awakened in adulthood? This requires us to shift our research focus toward the epigenetic imprints established early in life. How do prenatal or neonatal environmental exposures and microbial colonization set a threshold for skin immune cells, making them more easily triggered to reactivate fetal programs later in life (139)? Longitudinal studies utilizing spatial and single-cell technologies will be crucial for tracking the dynamics of immune cell populations throughout skin development and in the progression of disease. Whether future therapies can target specific epigenetic regulators—forcing those macrophages or T cells currently in a fetal-like inflammatory state to re-differentiate back to a healthy adult homeostatic state—represents a promising avenue for treating developmental skin disorders and promoting effective skin regeneration. Finally, understanding the role of the skin microbiome in modulating immune-tissue crosstalk during skin development and its implications for overall skin health and disease is an area that warrants further exploration.
